# *JAK2* Exon 14 Skipping in Patients with Primary Myelofibrosis: A Minor Splice Variant Modulated by the JAK2-V617F Allele Burden

**DOI:** 10.1371/journal.pone.0116636

**Published:** 2015-01-24

**Authors:** Paolo Catarsi, Vittorio Rosti, Giacomo Morreale, Valentina Poletto, Laura Villani, Roberto Bertorelli, Matteo Pedrazzini, Michele Zorzetto, Giovanni Barosi, AGIMM investigators

**Affiliations:** 1 Center for the Study and Treatment of Myelofibrosis, Biotechnology Research Laboratories, Fondazione IRCCS “Policlinico San Matteo”, Pavia (PV), Italy; 2 Viticulture Research Center, Consiglio per la Ricerca e la sperimentazione in Agricoltura, Conegliano (TV), Italy; 3 Laboratory of Biomolecular Sequence and Structure Analysis for Health, Fondazione “Bruno Kessler”, Trento (TN), Italy; 4 Cardiovascular Genetics Laboratory, Biomedical and Technology Research Centre, Istituto Auxologico Italiano, Cusano Milanino (MI), Italy; 5 Laboratory of Biochemistry and Genetics, Division of Pneumology, Department of Molecular Medicine, Fondazione IRCCS “Policlinico San Matteo”, Pavia (PV), Italy; Cancer Research Centre of Lyon, FRANCE

## Abstract

**Background:**

Primary myelofibrosis (PMF) is an acquired clonal disease of the hematopoietic stem cell compartment, characterized by bone marrow fibrosis, anemia, splenomegaly and extramedullary hematopoiesis. About 60% of patients with PMF harbor a somatic mutation of the *JAK2* gene (JAK2-V617F) in their hematopoietic lineage. Recently, a splicing isoform of *JAK2*, lacking exon 14 (JAK2Δ14) was described in patients affected by myeloproliferative diseases.

**Materials and Methods:**

By using a specific RT-qPCR method, we measured the ratio between the splicing isoform and the *JAK2* full-length transcript (JAK2+14) in granulocytes, isolated from peripheral blood, of forty-four patients with PMF and nine healthy donors.

**Results:**

We found that JAK2Δ14 was only slightly increased in patients and, at variance with published data, the splicing isoform was also detectable in healthy controls. We also found that, in patients bearing the JAK2-V617F mutation, the percentage of mutated alleles correlated with the observed increase in JAK2Δ14. Homozygosity for the mutation was also associated with a higher level of JAK2+14. Bioinformatic analysis indicates the possibility that the G>T transversion may interfere with the correct splicing of exon 14 by modifying a splicing regulatory sequence.

**Conclusions:**

Increased levels of *JAK2* full-length transcript and a small but significant increase in *JAK2* exon 14 skipping, are associated with the JAK2-V617F allele burden in PMF granulocytes. Our data do not confirm a previous claim that the production of the JAK2Δ14 isoform is related to the pathogenesis of PMF.

## Introduction

The human *JAK2* gene occupies a genomic region of about 14 kilobases (kb) on the short arm of chromosome 9 (9p24.1); it produces a transcript of 5.3 kb consisting of 25 exons that is translated into a cytoplasmic tyrosine kinase of 1132 amino acids, and belongs to the Janus kinase family (NCBI gene ID: 3717). In myeloproliferative neoplasms (MPNs), a somatic guanine-thymine substitution (c.1849G>T) located in the terminal part of exon 14 of *JAK2*, has been identified [1–4]. The consequent amino acid change, valine 617 to phenylalanine (JAK2-V617F), alters the structure of the pseudokinase domain with important consequences in activation [5–7]. This mutation is observed in almost all patients with polycythemia vera and in more than half of those with essential thrombocythemia or primary myelofibrosis (PMF). The measure of the ratio between mutated and total alleles in genomic DNA extracted from granulocytes (usually designated as “JAK2-V617F allele burden”) is used either at diagnosis for prognostic information or during treatment as a means to assess minimal residual disease [8].

By using the quantitative fragment length analysis technique, Ma *et al*. [9] described an alternative splicing event in the *JAK2* gene, resulting in the missing exon 14 both in plasma and in granulocytes of patients with MPNs. The transcript was found in ratios ranging from 2% to 26% compared to the amount of the full-length isoform, and it was reported to be translated into a truncated protein of approximately 70 kDa. As it was detected only in patients with MPNs, and more likely in patients tested negative for JAK2-V617F, it was suggested that the isoform could play a significant role in the pathophysiology of MPNs. The authors hypothesized that the truncated protein isoform dimerizes with the wild type JAK2, activating its kinase domain and consequently the JAK2-STAT pathway.

In this study, we assessed the exon 14-skipping variant (JAK2Δ14) in granulocytes of patients with PMF by using an isoform specific RT-qPCR method (S1, S2 Figs.) [10]. Moreover, we investigated the possible mechanism driving the alteration of splicing associated with the JAK2-V617F mutation.

## Materials and Methods

### Ethics statement

All work was performed according to a protocol approved by the Ethic Committee of the IRCCS Policlinico S. Matteo Foundation. Written informed consent was obtained from each patient before data were entered in the database.

### Patients and samples

We tested peripheral blood samples of 44 patients with PMF selected from those referred to the Center for the Study of Myelofibrosis at the Fondazione IRCCS Policlinico S. Matteo. The diagnosis of PMF was based on 2008 WHO criteria [11]. Fourteen patients were JAK2-V617F negative (wild type), and thirty positive for the V617F mutation (a cutoff of 1% mutated alleles was fixed as positive). In addition, we tested nine healthy control individuals.

The samples were collected using 0.105 M sodium citrate tubes (BD Vacutainer), stored at 4°C and processed within 4 hours after collection. Blood granulocytes were isolated from the lower interface of a Lympholyte-H density gradient (1.077 g/cm^3^ at 20°C, Cedarlane Laboratories Ltd.) and then submitted to erythrocyte lysis (BD Pharm Lyse, BD Biosciences).

Both DNA and RNA were extracted from granulocytes and cell lines (see subsequent paragraph “Cell line culture”). Total RNA was extracted with the miRNeasy Mini Kit (Qiagen) and further DNA purified by on-column digestion with the RNase-free DNase Set (Qiagen), according to the manufacturer’s instructions. Genomic DNA was extracted using the QIAamp DNA Blood Mini Kit (Qiagen). Nucleic acids were quantified with a Nanodrop 1000 spectrophotometer (Thermo Scientific).

cDNA synthesis was carried out using the iScript kit (Bio-Rad). In brief, 150 ng of each total RNA sample was reverse transcribed using a blend of oligo-dT and random primers, subsequently diluted with nuclease–free water to 3.75 ng/*μ*L (total RNA equivalent) and stored at -80°C.

The quality of RNAs extracted from granulocytes and cell lines was assessed in two healthy individuals, four patients and one cell line, randomly chosen (S1 Table). The cDNAs resulting from reverse transcription of these RNA samples were analyzed, using qPCR methods, in order to test the following parameters: (i) RNA integrity, using a 5′/3′ ratio mRNA integrity assay [12] (qHsaCtlD0001002, Bio-Rad); (ii) contamination from genomic DNA, using an assay that targets an untranscribed region of the human genome (qHsaCtlD0001004, Bio-Rad); (iii) presence of PCR inhibitors, using a positive PCR control assay that targets a synthetic DNA added to the reaction mix (qHsaCtlD0001003, Bio-Rad).

### Cell line culture

DAMI and K562 cell lines were cultured from laboratory stocks, while the UKE-1 cell line was generously provided by the original investigators [13]. Cells were routinely cultured in Iscove Modified Dulbecco’s Medium [IMDM] supplemented with 10% fetal bovine serum, 2% glutamine and 1% penicillin/streptomycin, at 37°C in a fully humidified incubator in the presence of 5% CO2. Where indicated, cycloheximide (CHX) (CAS 66-81-9, 25 mg/mL in DMSO) was added to the medium at a final concentration of 10 *μ*g/mL, 8 hours before harvesting. Three independent experiments for each condition were performed using the same cell lines.

### RT-qPCR gene expression analysis

Primers for EvaGreen assays (S2 Table) were designed using the Beacon Designer 7.9 software (Premier Biosoft International). Quantification of transcripts was carried out in a 15 *μ*L reaction mix containing 1X SsoFast EvaGreen Supermix (Bio-Rad) and 400 nM of each primer. The PCR conditions were 95°C for 30 sec followed by 40 cycles of 95°C for 5 sec and 60°C for 5 sec. Melting curves were generated after amplification in the range 65–95°C with increments of 0.2°C every 10 sec. For each experiment, 3 *μ*L of cDNA (corresponding to 11.3 ng of total RNA) were used. The PCR data were collected using the CFX96 Real-Time System (Bio-Rad). Each sample was tested in duplicate. Calculation of normalized relative expression levels was done using the Qbase Plus software version 2 (Biogazelle): a three-point serial dilution (1:4) of a mix of cDNA from patients and controls was included for each gene, to perform the amplification efficiencies correction; three samples were included in each run to generate an inter-run calibration; normalization was performed using the most stably expressed reference gene (or the two most stable, in the case of *in vitro* experiments) which was selected using the geNorm algorithm [14], with the following candidates: *YWHAZ*, *GAPDH*, *HPRT1*, *UBC*. Other authors have validated, in nine human bone marrow samples, the expression stability of the above-mentioned reference genes [14].

### Standard curves

The percentage of JAK2Δ14 compared to the full-length isoform JAK2+14 was calculated using absolute standard curves. The PCR products corresponding to the full-lenght transcript and skipped isoform (S2 Table) were run on 2% agarose gels in TBE buffer. The amplified fragments were excised and purified from the gel using QIAquick spin columns (Qiagen). The concentrations of the PCR products were measured using both the Quantifluor dsDNA System on a Quantifluor-ST fluorometer (Promega) and the Nanodrop 1000 spectrophotometer (Thermo Scientific). The molecular weight of the PCR fragments was determined using the software MacVector (MacVector, Inc.) and used for the conversion of micrograms to picomoles. Finally, equimolar dilutions of PCR fragments were used to generate the standard curves (S2 Fig.).

### JAK2-V617F allele burden in genomic DNA and cDNA

JAK2-V617F allele burden in the genomic DNA and cDNA obtained by retrotranscription of total-RNA from granulocytes was measured by allele-specific quantitative PCR, as previously described [15]. The JAK2-V617F allele burden was calculated by comparison with a standard curve obtained by a dilution series of genomic DNA from a patient with 100% allele burden into donor wild type DNA, in the following proportions: 2%, 5%, 12%, 25%, 50%, 75%, 95%, and 100%. To quantify the number of mutated transcripts, a similar standard curve was obtained by mixing the cDNA from a 100% mutated patient into donor wild type cDNA with identical *JAK2* expression levels. qPCR reactions were carried out in a 20 *μ*L reaction mix containing 1X SsoFast Probes Supermix (Bio-Rad), 400 nM of each primer and 200 nM of 6-FAM/BHQ-1 hydrolysis probe (Sigma-Aldrich). The PCR conditions were 95°C for 2 min followed by 40 cycles of 95°C for 5 sec and 62°C for 10 sec. For each experiment, 45 ng of genomic DNA or 11.3 ng of cDNA, were used.

### Reverse Trancription-PCR (RT-PCR) experiments

RT-PCR reactions (35 cycles, annealing temperature determined with a gradient between 50°C and 60°C, 1 min/kb extension) were performed in a C1000 thermal cycler (Bio-Rad) using granulocyte’s total RNA from patients and healthy individuals (see S3 Table for the list of the primers used). The amplifications were carried out using the GoTaq Flexi DNA Polymerase (Promega) according to the manufacturer’s instructions. The PCR products were run on a 2% agarose gel in TBE buffer and stained with 1x Sybr Gold (Invitrogen).

### Bioinformatic analysis

The Human Splicing Finder 3.0 and ESEfinder 3.0 web servers were used to analyze the *JAK2* exon 14 sequence to search for known exonic splicing enhancer and silencer (ESE/ESS) motifs, in wild type sequence and in the presence of the c.1849G>T (V617F) mutation. MacVector 12 (MacVector, Inc.) application was used for editing of *JAK2* sequences (NG_009904.1, NM_004972.3), open reading frame analysis, protein prediction and calculation of the molecular weight of PCR products.

### Statistical analysis

The relationship between measured variables was analyzed using bivariate linear regression and multiple regression analyses. The non-parametric Mann-Whitney *U* test was used to compare the variables measured in patients and controls. Fisher’s PLSD *post-hoc* test and Student’s unpaired *t*-test were used to study, respectively, pairwise differences in gene expression between cell lines and normal granulocytes and between *in vitro* experimental conditions. Differences were declared significant below the significance level of 0.05. StatView version 5.0.1 (SAS Institute Inc.) was used for statistical analysis. Box plots were used to represent the distributions of variables. In box plots, horizontal lines display 25^th^, 50^th^, 75^th^ percentiles and lowest and highest values of a variable. In scatter diagrams, dotted lines were used to represent the 95% confidence band of the regression line. Prism 4.0c (GraphPad Software Inc.) was used for data representation.

## Results

### JAK2Δ14 in healthy controls and PMF patients

Measurable levels of the spliced JAK2Δ14 variant were detected in blood granulocytes both in healthy controls and PMF patients. The median value of the JAK2Δ14 transcript, as expressed in percentage of the full-length transcript (JAK2+14), was higher in PMF patients than in controls without reaching statistical significance (0.71% vs. 0.42%, respectively; Mann-Whitney *U* test: *p* = 0.076).

**Fig 1:**
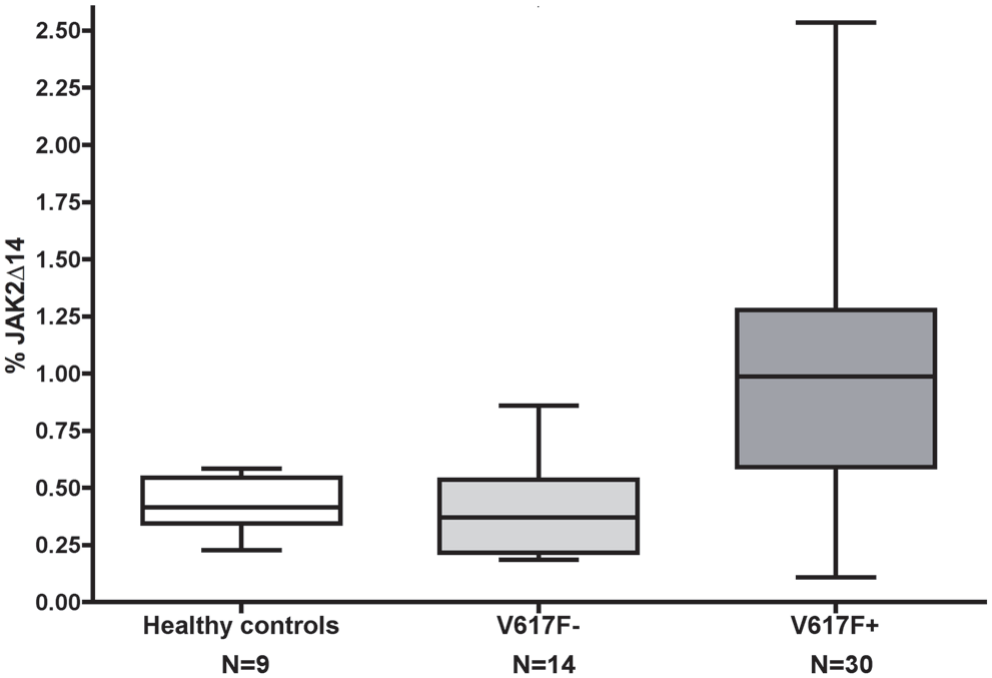
JAK2-617F positive patients have higher levels of JAK2Δ14 than wild type patients and healthy controls. Mann-Whitney *U* test: *p* < 0.001.

**Fig 2:**
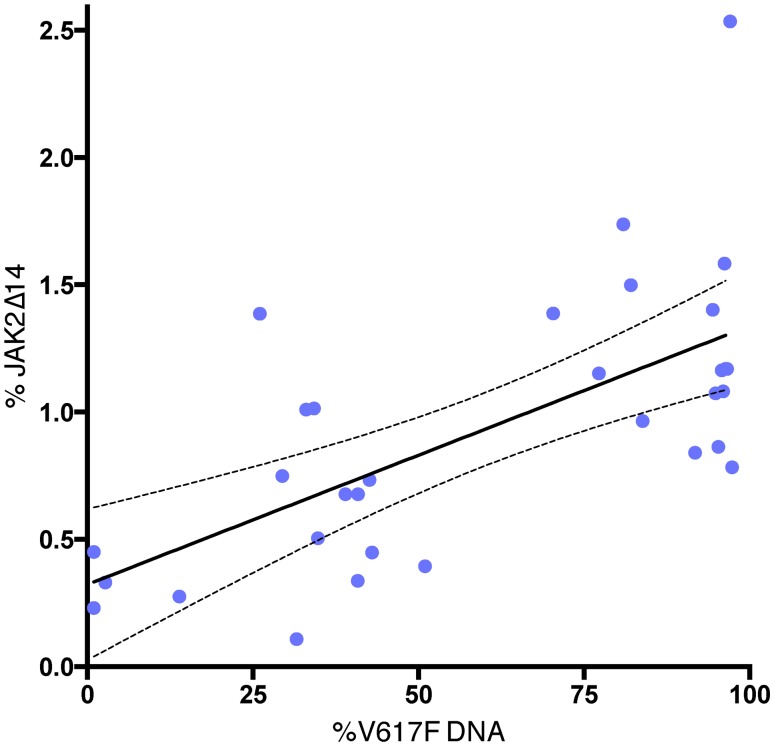
In PMF patients, levels of mRNA isoform JAK2Δ14 correlate with the percentage of JAK2-V617F mutated alleles. *R* ^2^ = 0.43, *p* < 0.001.

However, while wild type patients had JAK2Δ14 levels that did not differ significantly from healthy donors (0.37% vs. 0.42%, respectively; Mann-Whitney *U* test: *p* = 0.378; Fig. 1), patients carrying the JAK2-V617F mutation showed significantly greater percentages of the spliced isoform with respect to controls (median: 1.0%, interquartile range: 0.49; Mann-Whitney *U* test: *p* < 0.01; Fig. 1). Moreover, in JAK2-V617F mutated patients, the level of the isoform was strictly correlated with the JAK2-V617F allele burden (*R* ^2^ = 0.43, *p* < 0.001; Fig. 2).

### Bioinformatic analysis of *JAK2* exon 14

**Fig 3:**
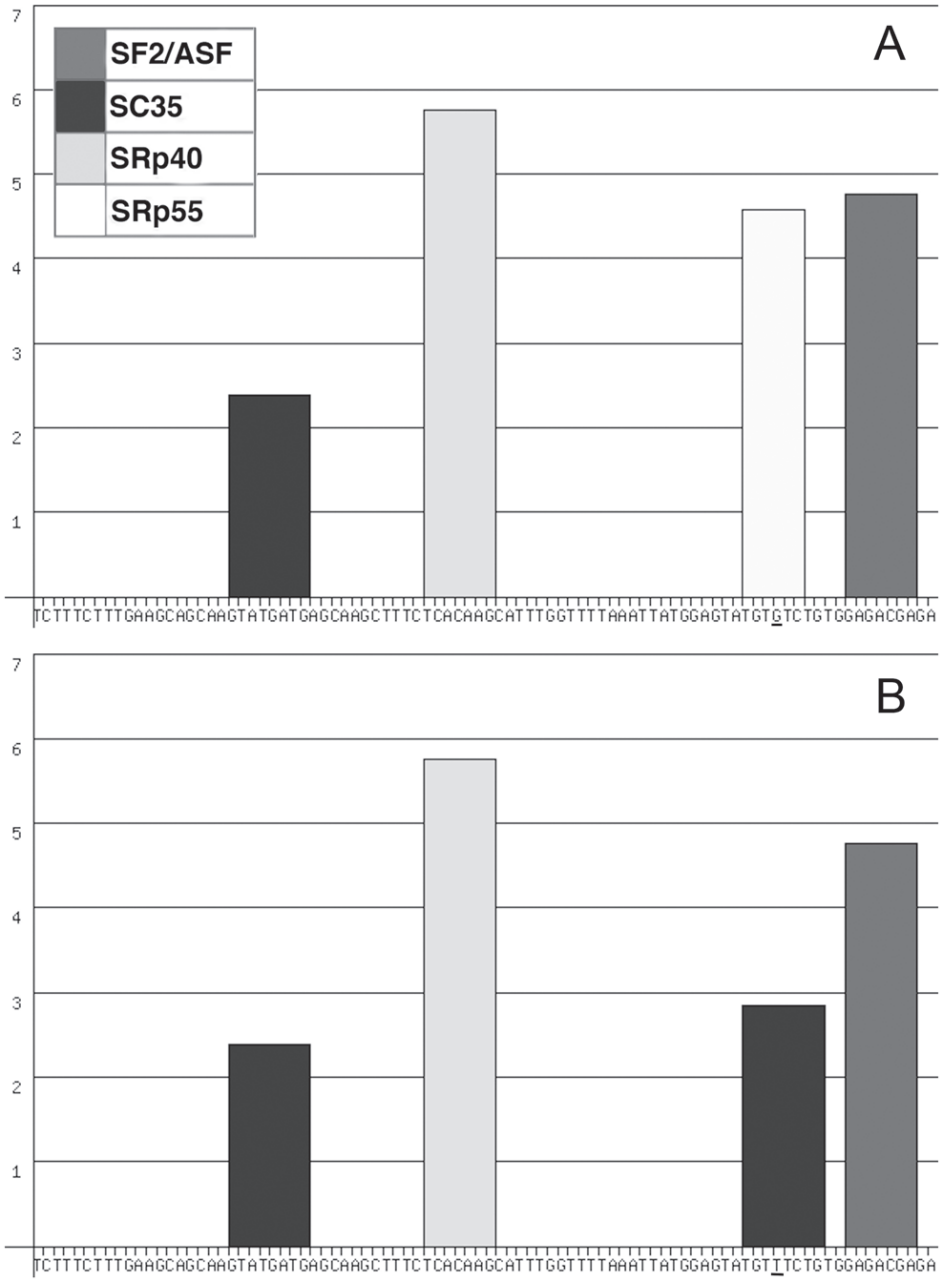
ESE finder analysis of wild type (a) and mutated (b) *JAK2* exon 14 sequences (88 nt). The default threshold values for SF2/ASF (SRSF1), SC35 (SRSF2), SRp40 (SRSF5) and SRp55 (SRSF6) were, respectively, 1.956, 2.383, 2.67 and 2.676. With the exception of SC35, the above-mentioned threshold values were increased by one unit in order to present only the best scores for each SR protein. The width of each bar reflects the length of the motif, the placement of each bar along the X-axis represents the position of a motif along the DNA sequence, the height of the bar represents the numerical score on the Y-axis. The G to T missense substitution (exon 14 position: 73rd nucleotide) affects the SRp55 binding motif TGTGTC, reducing the score from 4.58 to 2.28 (below the default threshold) and creating a sequence containing a potential SC35 binding motif (TGTTTCTG score: 2.843).

We analyzed the possible effects of the c.1849G>T mutation on exonic splicing regulatory sequences (ESE or ESS) using ESEfinder 3.0 [16] and Human Splicing Finder 3.0 [17] programs. The *JAK2* exon 14 consists of 88 base pairs and the mutation occurs at position 73. In the mutated sequence, two independent algorithms [16, 18] identified a possible alteration of an exonic ESE site. Another algorithm [19], designed to search for phylogenetically conserved sequences that can act as silencers or enhancers depending on exonic context, recognizes, in the same sequence identified by ESEfinder (TGTGTC) a possible splicing regulatory element disrupted by the mutation (Fig. 3, S4 Table). The ESEfinder 3.0 analysis also showed that this sequence is a nearly optimal consensus motif for SRp55 (TGCGTC). Two other computational approaches [20, 21] predict the potential creation of an exonic splicing silencer (ESS). Conversely, in the same exonic subsequence, the ESEfinder algorithm recognizes the possible creation of an ESE sequence containing a SC35 binding motif (Fig. 3, S4 Table). Another matrice, implemented in Human Splice Finder 3.0, indicates hnRNP-A1 as a ligand of a potential ESS (TATGTG), but the software predicted only a slight enhancing effect of the G>T transversion on its function (S4 Table).

### Regulation of *JAK2* transcription

**Fig 4:**
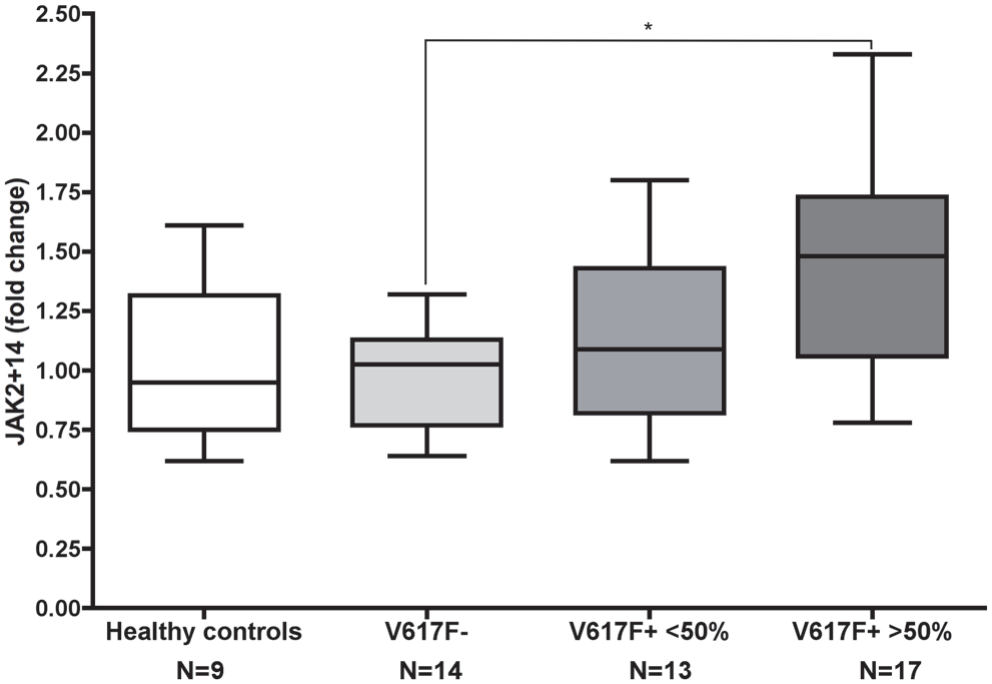
Box-plot chart representing the levels of *JAK2* major transcript (JAK2+14) in patients and controls. Quantities are expressed as fold changes compared to the mean quantity in healthy subjects. The levels of JAK2+14 are significantly higher in patients bearing the JAK2-V617F mutation in more than 50% of alleles (median: 1.46, interquartile range: 0.65) with respect to the wild type patients (median: 1.0, interquartile range: 0.36). Mann-Whitney *U* test: *p* < 0.01.

*YWHAZ* was used as a reference gene for expression studies in granulocytes because it was experimentally found to be the most stably expressed in these cells. In order to study the regulation of *JAK2* gene transcription, we analyzed the level of expression of *JAK2* full-length mRNA in patients with PMF and its relationship with the amount of the JAK2Δ14 splicing isoform. In agreement with previously reported data [22], the JAK2+14 transcript levels were significantly higher in patients with the highest V617F allele burden (Fig. 4). Indeed, we observed a median 50% increase of JAK2+14 in patients bearing the V617F mutation in more than 50% of alleles, compared to those with a wild type genotype (Mann-Whitney *U* test: *p* < 0.01).

Since the *JAK2* exon 14 skipping, changes the open reading frame and results in the introduction of a premature termination codon (PTC) (S4 Fig.), we wondered whether JAK2Δ14 could be the target of the nonsense-mediated mRNA decay (NMD) system that is known to require the presence of a PTC at more than 50–55 nucleotides (nt) from the last junction between exons [23, 24]. With RT-PCR, we documented that the JAK2Δ14 transcript extends at least over exon 18 (S5 Fig., S3 Table).

**Fig 5:**
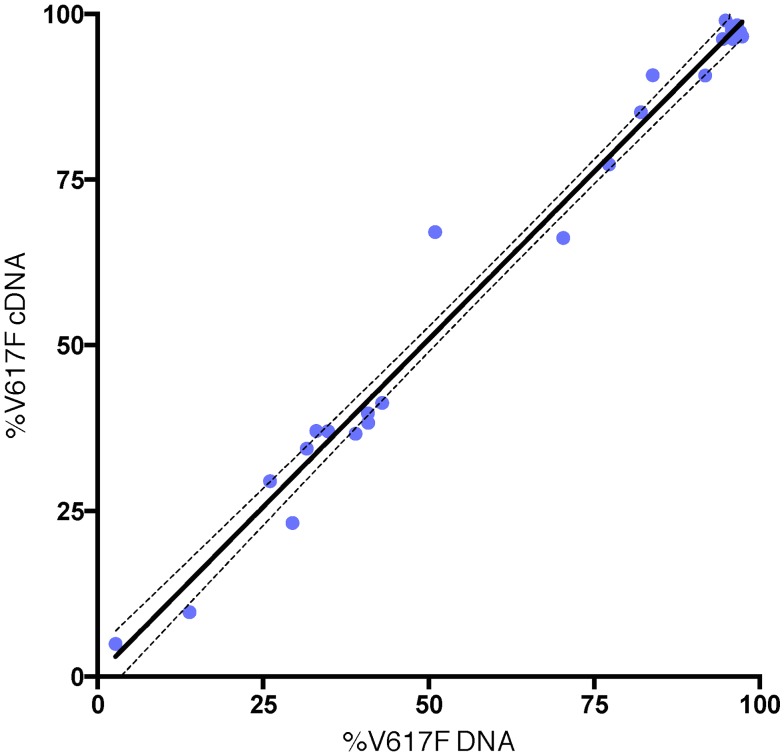
Regression analysis. Shows that the proportion of mutated alleles in the genomic DNA corresponds to the proportion of mutated transcripts (Y = 0.293 + 1.012 * *x*, *R* ^2^ = 0.983).

The percentage of mutated transcripts in cDNA was measured to evaluate the hypothesis that a combination of NMD activity and preferential production of the isoform by pre-mRNA containing the V617F mutation could lead to a decrease in production of JAK2+14 mutated transcripts in the samples positive for the JAK2-V617F mutation. Conversely, in agreement with another study [15], we observed that the proportion of JAK2-V617F mutated alleles, was the same for both genomic DNA and cDNA (Fig. 5).

### JAK2Δ14 in cell lines bearing the JAK2-V617F mutation

**Fig 6:**
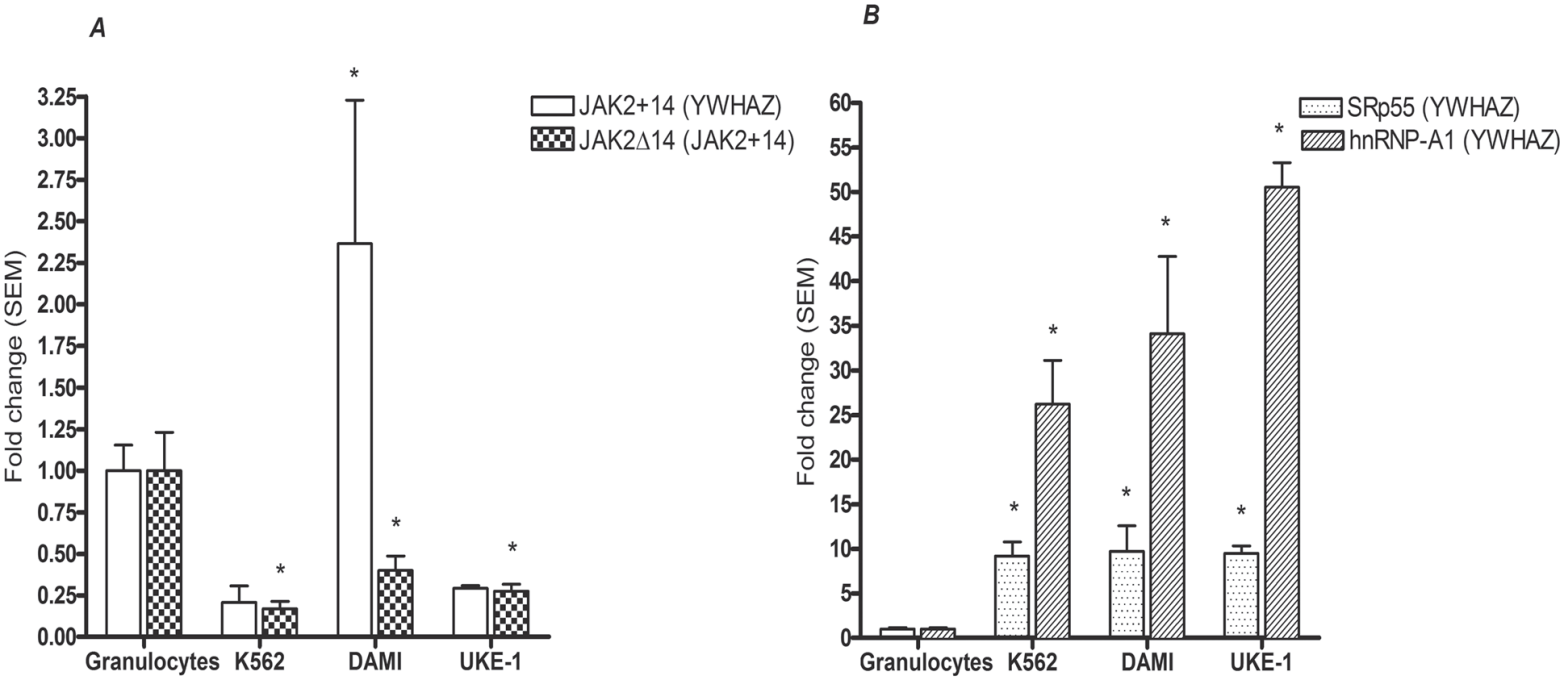
Transcript quantification of JAK2+14 and relative extent of JAK2Δ14 (*A*), SRp55 and hnRNP-A1 splicing factors (*B*) in cell lines either wild type (K562) or homozygous (UKE-1, DAMI) for the JAK2-V617F mutation. Quantities are expressed as fold changes compared to the mean quantity measured in healthy donor granulocytes. The data are means of transcript ratios (± SEM) of three independent experiments performed using the same cell lines (K562, DAMI and UKE-1) or four healthy individuals (granulocytes). Asterisks indicate significant changes in gene expression between cell line and normal granulocytes.

In order to assess the effect of the JAK2-V617F mutation on *JAK2* exon 14 skipping in cells other than granulocytes, we assayed the expression of *JAK2* main transcript (JAK2+14) and the relative level of JAK2Δ14 in cell lines either JAK2-V617F homozygous (UKE-1, DAMI) or wild type (K562) [25]. In K562 and UKE-1 lines, the expression of JAK2+14 was lower than that observed in normal granulocytes while in DAMI, the presence of many copies of the gene [25] caused mRNA levels that were more than two times higher than in normal granulocytes. Nevertheless, the relative amount of JAK2Δ14 in all three cell lines was lower than that measured in granulocytes: between 20 and 40% of the average value observed in granulocytes (Fig. 6a). The absence of an enhancing effect of the c.1849G>T mutation on the level of the exon 14-skipping isoform in the JAK2-V617F homozygous cell lines could be due to several factors. We tested the hypotheses that different concentrations of splicing factors in these cells and/or a higher degradation due to the NMD system may maintain JAK2Δ14 at low levels. To assess the first hypothesis, we measured the mRNA levels of two splicing factors indicated in bioinformatics analysis: SRp55 and hnRNP-A1. In all three cell lines, the levels of both mRNAs were vastly higher than those observed in granulocytes: about 10 times for SRp55 and between 26 and 50 times for hnRNP-A1 (Fig. 6b). To investigate the possibility of NMD system involvement, we treated the above-mentioned cell lines with CHX, a protein synthesis inhibitor that locks the NMD system activity [24]. To verify the effectiveness of the treatment we measured the expression of a *SRp55* splicing variant containing a PTC (SRp55-PTC+b). It has been demonstrated that the inhibition of the NMD system, both with CHX and through depletion of UPF1, causes an increase of this variant in HeLa cells [26]. Our experiment confirms the results obtained by Lareau *et al*. [26]. Eight hours after treatment, we observed a significant increase in the levels of SRp55-PTC+b messenger in all cell lines. On the contrary, neither the level of JAK2+14 nor that of JAK2Δ14, were significantly changed after treatment with CHX (S3 Fig.).

## Discussion

Besides affecting the amino acid sequence, which in turn is critical for the function of the protein, missense and nonsense mutations can also alter splicing regulatory sequences, that lead to an incorrectly spliced transcript [27, 28].

With this study we characterized an exon 14-skipping isoform of the *JAK2* gene that is mutated in approximately 60% of patients with PMF. We found that *JAK2* exon 14 skipping occurs constitutively both in healthy individuals and PMF patients. In PMF patients bearing the JAK2-V617F mutation, the production of the skipped isoform correlated with the percentage of mutated alleles. This observation, combined with the results of bioinformatic analysis of the *JAK2* exon 14 sequence, allowed us to hypothesize that the c.1849G>T somatic transversion, in addition to determining the amino acid substitution p.V617F, could change a splicing regulatory sequence, causing an increase in the production of the skipping isoform in mutated subjects. However, even in the presence of high JAK2-V617F allele burden, the amount of isoform represented no more than 2.5 percent of the full-length transcript. Therefore, having found some evidence that JAK2Δ14 could meet the criteria as the target of NMD, we asked whether this system intervenes by degrading the isoform and consequently, minimizing the potential damage due to a hypothetical abundant production of JAK2Δ14 caused by the JAK2-V617F mutation.

As a matter of fact, in-frame nonsense codons located upstream of the last junction between exons were recognized as PTCs and targeted the mRNA for degradation. Nevertheless, a study by Pan *et al*. [29] showed that the majority of transcripts containing PTCs generated by alternative splicing (which accounts for about one third of the splice variants), are present at low levels, and that only a small fraction of these is regulated by the NMD system. It is not clear to what extent such variants are functionally relevant, but a recent deep sequencing analysis of the human lymphoblastoid cell transcriptome [30] seemed to confirm the hypothesis that a large fraction may arise as a consequence of the probabilistic nature of the splice sites recognition, and can be classified as non-functional “noise” [31]. Based on the above-mentioned results and on the analysis of the percentage of the c.1849G>T mutated alleles in cDNA compared to genomic DNA, we infer that the overproduction of the isoform might be minimal. The absence of a significant effect of the increased production of JAK2Δ14 on the expression of the mutated alleles, led us to conclude that the observed low level of this splice variant was probably due to its limited production rather than to a massive degradation operated by the NMD system. Indeed, we could not detect any significant enhancement in the levels of JAK2Δ14 following NMD inhibition with CHX in model cell lines.

In order to explain why the presence of a homozygous mutation does not affect the production of JAK2Δ14 in DAMI and UKE-1 cells, we proposed that a different concentration of splicing factors in these cell lines could maintain JAK2Δ14 at low levels. Indeed, the transcript levels of hnRNP-A1 and SRp55 (i.e. two splicing factor suggested by the bioinformatics analysis) are one order of magnitude higher in cell lines compared to their expression levels in granulocytes. Previous studies showed that altered concentrations of SRp55 and hnRNP-A1 determine quantitative changes in the ratio between isoforms of cancer related genes [32–34]. Assuming that the levels of hnRNP-A1 and SRp55 mRNAs are directly proportional to the amount of the related splicing factors, we tested two alternative hypotheses. The first was that the mutation increases the binding capacity of an ESS bound by the factor hnRNP-A1. This conjecture is contradicted by what was observed in the cell lines above, where the high levels of hnRNP-A1 should lead to higher activity of the linked ESS and a consequential increase in JAK2Δ14 levels. The second was that the mutation disrupts an ESE linked by the SRp55 protein. This hypothesis is compatible with our observations because the high levels of SRp55 in DAMI and UKE-1 cells could compensate for the predicted interference caused by the c.1849G>T mutation with the binding of this factor. These findings together with the above-discussed results, although not sufficient to derive definitive conclusions, support the initial hypothesis that the mutation interferes with the splicing of exon 14 through the modification of a splicing regulatory sequence. Further experiments are needed to confirm this hypothesis and to analyze the different possibilities that emerged from computational analysis.

Another result of this study is that the JAK2-V617F mutation was also associated with an increased amount of full-length isoform JAK2+14. Also in this case, the effect was proportional to the percentage of mutated alleles and in homozygous patients consisted in an average 50% increase of JAK2+14 levels. Although our data do not allow clarification of the mechanism that determines the increase in transcript levels, this observation may support a previously proposed hypothesis raised to explain why the individuals carrying the 46/1 haplotype have an increased risk of developing the mutation [35]. In accordance with the “fertile ground” hypothesis [36], the mutation occurs with the same probability on the different alleles, but the cells in which the mutation occurs on 46/1 haplotype have a selective advantage. It can be hypothesized that the observed increment in *JAK2* mRNA levels is caused by the occurrence of the JAK2-V617F mutation on the 46/1 haplotype. In this case, the increased production of the mutant JAK2 protein could contribute to the above-mentioned selective advantage.

Our approach did not confirm the presence of high amounts of JAK2Δ14 observed by Ma *et al*. [9]. This could be due to the fact that the Quantitative Fragment Length Analysis technique, originally developed for the prenatal diagnosis of chromosomal abnormalities [37], used by Ma *et al*., is less suitable for the quantification of splice variants. Since with this method, fragments of different sizes are simultaneously amplified, overestimation of the amount of the isoform that produces a shorter fragment is possible because it tends to be amplified preferentially with respect to the full-length counterpart. Moreover, if the amplification is not limited to the exponential phase, the least represented isoform is overestimated [37–39].

The experimental evidence described here argues against the hypothesis that the presence of this splice variant could be pathogenetically associated with MPNs. It is unlikely that the slight increase in the amount of JAK2Δ14 could produce a truncated protein at significant levels. In addition, the fact that the level of JAK2Δ14 is comparable in healthy subjects and in patients is in contrast with the hypothesis that its presence could be involved in the pathogenesis of PMF. Moreover, it was observed that the ectopic expression of a truncated protein isoform of JAK2 lacking the protein kinase domain (Jak2-829), has the effect of blocking the erythropoietin-dependent inhibition of apoptosis [40]. It can be hypothesized from the above observation that the production of a truncated protein isoform of JAK2, resulting from translation of JAK2Δ14, could have an antiproliferative effect that would be desirable in MPNs.

## Supporting Information

S1 Fig JAK2Δ14 RT-qPCR analysis in healthy controls and PMF patients. EvaGreen amplification signals for YWHAZ, JAK2+14 and JAK2Δ14, in two individuals with normal (grey) and increased (black) level of the exon 14-skipping isoform. Top left box shows melting peaks obtained by High Resolution Melting Analysis of the three amplification products: it can be observed the different melting peak morphology caused by the JAK2-V617F mutation present in the JAK2+14 transcripts of the patient with increased level of JAK2Δ14.
(JPG)

S2 Fig Quantification of PCR-JAK2+14 and PCR-JAK2Δ14 by absolute standard curves. Equimolar dilutions of PCR-JAK2Δ14 (left) and PCR-JAK2+14 (right) amplicons, were used to generate two standard curves utilized to calculate the percentage of alternative transcript. The three points correspond to 1:4 serial dilutions of the gel-purified PCR products.
(JPG)

S3 Fig Effect of CHX treatment on *JAK2* alternative transcripts containing PTCs. RT–qPCR was used to assay mRNAs levels in cell lines either homozygous for the JAK2-V617F mutation (UKE-1, DAMI) or wild type (K562). Transcript level ratios between CHX-treated (orange) and untreated cells (blue), are shown for: *SRp55* constitutive transcript (SRp55), *SRp55* PTC-containing isoform (SRp55-PTC+b), *JAK2* full-length transcript (JAK2+14) and *JAK2* exon 14 skipping isoform (JAK2Δ14). Data are expressed as means (± SEM) of three independent experiments performed using the same cell line. Normalized expression of targets genes was obtained using the two genes with the lowest geNorm M-value: *YWHAZ*/*HPRT1* for DAMI, *GAPDH*/*HPRT1* for K562 and UKE-1. Asterisks (*) indicate significant changes in gene expression after treatment.
(JPG)

S4 Fig Hypothetical translations of the JAK2Δ14 subsequence resulting from the junction between exons 13 and 15. The sense strand (black), its complementary strand (blue) and their possible phases of translation, are shown. Single-letter code is used to represent the amino acids. A stop codon is indicated by an asterisk (*). The reading frame, used in the translation of the full-length transcript (JAK2+14), is represented in the first row above the sense strand.
(JPG)

S5 Fig The alternative transcript (JAK2Δ14) extends at least until exon 18 and can be the target of the Nonsense Mediated Decay (NMD) system. (A) The diagram shows the location of the primers in the JAK2 full-length mRNA (above) and in the isoform lacking exon 14 (below). As in the qPCR, forward primers were specific for each isoform while the reverse primer was, in both amplifications, localized in exon 18 (S2 Table). In the alternative isoform, the hypothetical position of the stop codon and exon junction complexes (which is expected to activate the NMD system), are indicated. (B) Electrophoresis of PCR products obtained by amplifying the cDNA of a patient with 2.5% level of JAK2Δ14 isoform, at three different annealing temperatures. The expected amplicon sizes are 495 bp for the JAK2Δ14 isoform (PCR-Δex14/ex18) and 556 bp for the JAK2+14 constitutive isoform (PCR-ex14/ex18).
(JPG)

S1 Table RNA quality control assay results for seven randomly chosen cDNA samples used in this study. (PDF)

S2 Table Primers used in RT-qPCR experiments. (PDF)

S3 Table Primers used in RT-PCR experiments. (PDF)

S4 Table Identification of potential splicing regulatory sequences in wild type and V617F mutated *JAK2* exon 14 sequences. Twelve different matrices implemented in Human Splicing Finder (HSF) 3.0, are presented.
(PDF)

## References

[1] James C, Ugo V, Le Couédic J-P, Staerk J, Delhommeau F, et al (2005) A unique clonal JAK2 mutation leading to constitutive signalling causes polycythaemia vera. Nature 434: 1144–1148. 10.1038/nature03546 15793561

[2] Kralovics R, Passamonti F, Buser AS, Teo S-S, Tiedt R, et al (2005) A gain-of-function mutation of JAK2 in myeloproliferative disorders. N Engl J Med 352: 1779–1790. 10.1056/NEJMoa051113 15858187

[3] Baxter EJ, Scott LM, Campbell PJ, East C, Fourouclas N, et al (2005) Acquired mutation of the tyrosine kinase JAK2 in human myeloproliferative disorders. Lancet 365: 1054–1061. 10.1016/S0140-6736(05)71142-9 15781101

[4] Levine RL, Wadleigh M, Cools J, Ebert BL, Wernig G, et al (2005) Activating mutation in the tyrosine kinase JAK2 in polycythemia vera, essential thrombocythemia, and myeloid metaplasia with myelofibrosis. Cancer Cell 7: 387–397. 10.1016/j.ccr.2005.03.023 15837627

[5] Cross NCP (2011) Genetic and epigenetic complexity in myeloproliferative neoplasms. Hematology Am Soc Hematol Educ Program 2011: 208–214. 10.1182/asheducation-2011.1.208 22160036

[6] Tefferi A (2011) Mutations galore in myeloproliferative neoplasms: Would the real Spartacus please stand up? Leukemia 25: 1059–1063. 10.1038/leu.2011.92 21750560

[7] Kralovics R (2008) Genetic complexity of myeloproliferative neoplasms. Leukemia 22: 1841–1848. 10.1038/leu.2008.233 18754034

[8] Bench AJ, White HE, Foroni L, Godfrey AL, Gerrard G, et al (2013) Molecular diagnosis of the myeloproliferative neoplasms: UK guidelines for the detection of JAK2 V617F and other relevant mutations. Br. J. Haematol. 160: 25–34. 10.1111/bjh.12075 23057517

[9] Ma W, Kantarjian H, Zhang X, Wang X, Zhang Z, et al (2010) JAK2 exon 14 deletion in patients with chronic myeloproliferative neoplasms. PLoS ONE 5: e12165 10.1371/journal.pone.0012165 20730051 PMC2921382

[10] Vandenbroucke II, Vandesompele J, Paepe AD, Messiaen L (2001) Quantification of splice variants using real-time PCR. Nucleic Acids Res 29: E68–8. 10.1093/nar/29.13.e68 11433044 PMC55792

[11] Tefferi A, Vardiman JW (2008) Classification and diagnosis of myeloproliferative neoplasms: the 2008 World Health Organization criteria and point-of-care diagnostic algorithms. Leukemia 22: 14–22. 10.1038/sj.leu.2404955 17882280

[12] Nolan T, Hands R, Bustin S (2006) Quantification of mRNA using real-time RT-PCR. Nat Protoc 1: 1559–1582. 10.1038/nprot.2006.236 17406449

[13] Fiedler W, Henke RP, Ergün S, Schumacher U, Gehling UM, et al (2000) Derivation of a new hematopoietic cell line with endothelial features from a patient with transformed myeloproliferative syndrome: a case report. Cancer 88: 344–351. 10.1002/(SICI)1097-0142(20000115)88:2<344::AID-CNCR14>3.0.CO;2-6 10640966

[14] Vandesompele J, De Preter K, Pattyn F, Poppe B, Van Roy N, et al (2002) Accurate normalization of real-time quantitative RT-PCR data by geometric averaging of multiple internal control genes. Genome Biol 3: RESEARCH0034 10.1186/gb-2002-3-7-research0034 12184808 PMC126239

[15] Lippert E, Boissinot M, Kralovics R, Girodon F, Dobo I, et al (2006) The JAK2-V617F mutation is frequently present at diagnosis in patients with essential thrombocythemia and polycythemia vera. Blood 108: 1865–1867. 10.1182/blood-2006-01-013540 16728702

[16] Cartegni L, Wang J, Zhu Z, Zhang MQ, Krainer AR (2003) ESEfinder: A web resource to identify exonic splicing enhancers. Nucleic Acids Res 31: 3568–3571. 10.1093/nar/gkg616 12824367 PMC169022

[17] Desmet FO, Hamroun D, Lalande M, Collod-Beroud G, Claustres M, et al (2009) Human Splicing Finder: an online bioinformatics tool to predict splicing signals. Nucleic Acids Res 37: e67–e67. 10.1093/nar/gkp215 19339519 PMC2685110

[18] Zhang C, Li W-H, Krainer AR, Zhang MQ (2008) RNA landscape of evolution for optimal exon and intron discrimination. Proc Natl Acad Sci USA 105: 5797–5802. 10.1073/pnas.0801692105 18391195 PMC2311341

[19] Goren A, Ram O, Amit M, Keren H, Lev-Maor G, et al (2006) Comparative analysis identifies exonic splicing regulatory sequences—The complex definition of enhancers and silencers. Molecular Cell 22: 769–781. 10.1016/j.molcel.2006.05.008 16793546

[20] Wang Z, Rolish ME, Yeo G, Tung V, Mawson M, et al (2004) Systematic identification and analysis of exonic splicing silencers. Cell 119: 831–845. 10.1016/j.cell.2004.11.010 15607979

[21] Zhang XH-F, Chasin LA (2004) Computational definition of sequence motifs governing constitutive exon splicing. Genes Dev. 18: 1241–1250. 10.1101/gad.1195304 15145827 PMC420350

[22] Spasovski V, Tosic N, Nikcevic G, Stojiljkovic M, Zukic B, et al (2012) The influence of novel transcriptional regulatory element in intron 14 on the expression of Janus kinase 2 gene in myeloproliferative neoplasms. J Appl Genetics 54: 21–26. 10.1007/s13353-012-0125-x 23188718

[23] Kim VN, Kataoka N, Dreyfuss G (2001) Role of the nonsense-mediated decay factor hUpf3 in the splicing-dependent exon-exon junction complex. Science 293: 1832–1836. 10.1126/science.1062829 11546873

[24] Ishigaki Y, Li X, Serin G, Maquat LE (2001) Evidence for a pioneer round of mRNA translation: mRNAs subject to nonsense-mediated decay in mammalian cells are bound by CBP80 and CBP20. Cell 106: 607–617. 10.1016/S0092-8674(01)00475-5 11551508

[25] Quentmeier H, MacLeod RAF, Zaborski M, Drexler HG (2006) JAK2 V617F tyrosine kinase mutation in cell lines derived from myeloproliferative disorders. Leukemia 20: 471–476. 10.1038/sj.leu.2404081 16408098

[26] Lareau LF, Inada M, Green RE, Wengrod JC, Brenner SE (2007) Unproductive splicing of SR genes associated with highly conserved and ultraconserved DNA elements. Nature 446: 926–929. 10.1038/nature05676 17361132

[27] Cartegni L, Chew SL, Krainer AR (2002) Listening to silence and understanding nonsense: exonic mutations that affect splicing. Nat Rev Genet 3: 285–298. 10.1038/nrg775 11967553

[28] Holbrook JA, Neu-Yilik G, Hentze MW, Kulozik AE (2004) Nonsense-mediated decay approaches the clinic. Nat Genet 36: 801–808. 10.1038/ng1403 15284851

[29] Pan Q, Saltzman AL, Kim YK, Misquitta C, Shai O, et al (2006) Quantitative microarray profiling provides evidence against widespread coupling of alternative splicing with nonsense-mediated mRNA decay to control gene expression. Genes Dev. 20: 153–158. 10.1101/gad.1382806 16418482 PMC1356107

[30] Pickrell JK, Pai AA, Gilad Y, Pritchard JK (2010) Noisy Splicing Drives mRNA Isoform Diversity in Human Cells. PLoS Genet. 6: e1001236 10.1371/journal.pgen.1001236 21151575 PMC3000347

[31] Chern T-M, van Nimwegen E, Kai C, Kawai J, Carninci P, et al (2006) A simple physical model predicts small exon length variations. PLoS Genet. 2: e45 10.1371/journal.pgen.0020045 16683028 PMC1449888

[32] Filippov V, Schmidt EL, Filippova M, Duerksen-Hughes PJ (2008) Splicing and splice factor SRp55 participate in the response to DNA damage by changing isoform ratios of target genes. Gene 420: 34–41. 10.1016/j.gene.2008.05.008 18571879 PMC2562212

[33] Jensen MA, Wilkinson JE, Krainer AR (2014) Splicing factor SRSF6 promotes hyperplasia of sensitized skin. Nat. Struct. Mol. Biol. 21: 189–197. 10.1038/nsmb.2756 24440982 PMC4118672

[34] Bonomi S, di Matteo A, Buratti E, Cabianca DS, Baralle FE, et al (2013) HnRNP A1 controls a splicing regulatory circuit promoting mesenchymal-to-epithelial transition. Nucleic Acids Res 41: 8665–8679. 10.1093/nar/gkt579 23863836 PMC3794575

[35] Jones AV, Chase A, Silver RT, Oscier D, Zoi K, et al (2009) JAK2 haplotype is a major risk factor for the development of myeloproliferative neoplasms. Nat Genet 41: 446–449. 10.1038/ng.334 19287382 PMC4120192

[36] Campbell PJ (2009) Somatic and germline genetics at the JAK2 locus. Nat Genet 41: 385–386. 10.1038/ng0409-385 19338077

[37] Mansfield ES (1993) Diagnosis of Down syndrome and other aneuploidies using quantitative polymerase chain reaction and small tandem repeat polymorphisms. Hum. Mol. Genet. 2: 43–50. 10.1093/hmg/2.1.43 8490622

[38] Cirigliano V, Ejarque M, Fuster C, Adinolfi M (2002) X chromosome dosage by quantitative fluorescent PCR and rapid prenatal diagnosis of sex chromosome aneuploidies. Mol. Hum. Reprod. 8: 1042–1045. 10.1093/molehr/8.11.1042 12397218

[39] Rahil H, Solassol J, Philippe C, Lefort G, Jonveaux P (2002) Rapid detection of common autosomal aneuploidies by quantitative fluorescent PCR on uncultured amniocytes. Eur. J. Hum. Genet. 10: 462–466. 10.1038/sj.ejhg.5200833 12111640

[40] Zhuang H, Niu Z, He TC, Patel SV, Wojchowski DM (1995) Erythropoietin-dependent inhibition of apoptosis is supported by carboxyl-truncated receptor forms and blocked by dominant-negative forms of Jak2. J. Biol. Chem. 270: 14500–14504. 10.1074/jbc.270.24.14500 7782312

