# Supplementary figures and images for: JAK2 Exon 14 Skipping in Patients with Primary Myelofibrosis: A Minor Splice Variant Modulated by the JAK2-V617F Allele Burden
Source: PLoS One. 2015 Jan 24;10(1):e0116636. doi: 10.1371/journal.pone.0116636 (PMC4305294; doi:10.1371/journal.pone.0116636)

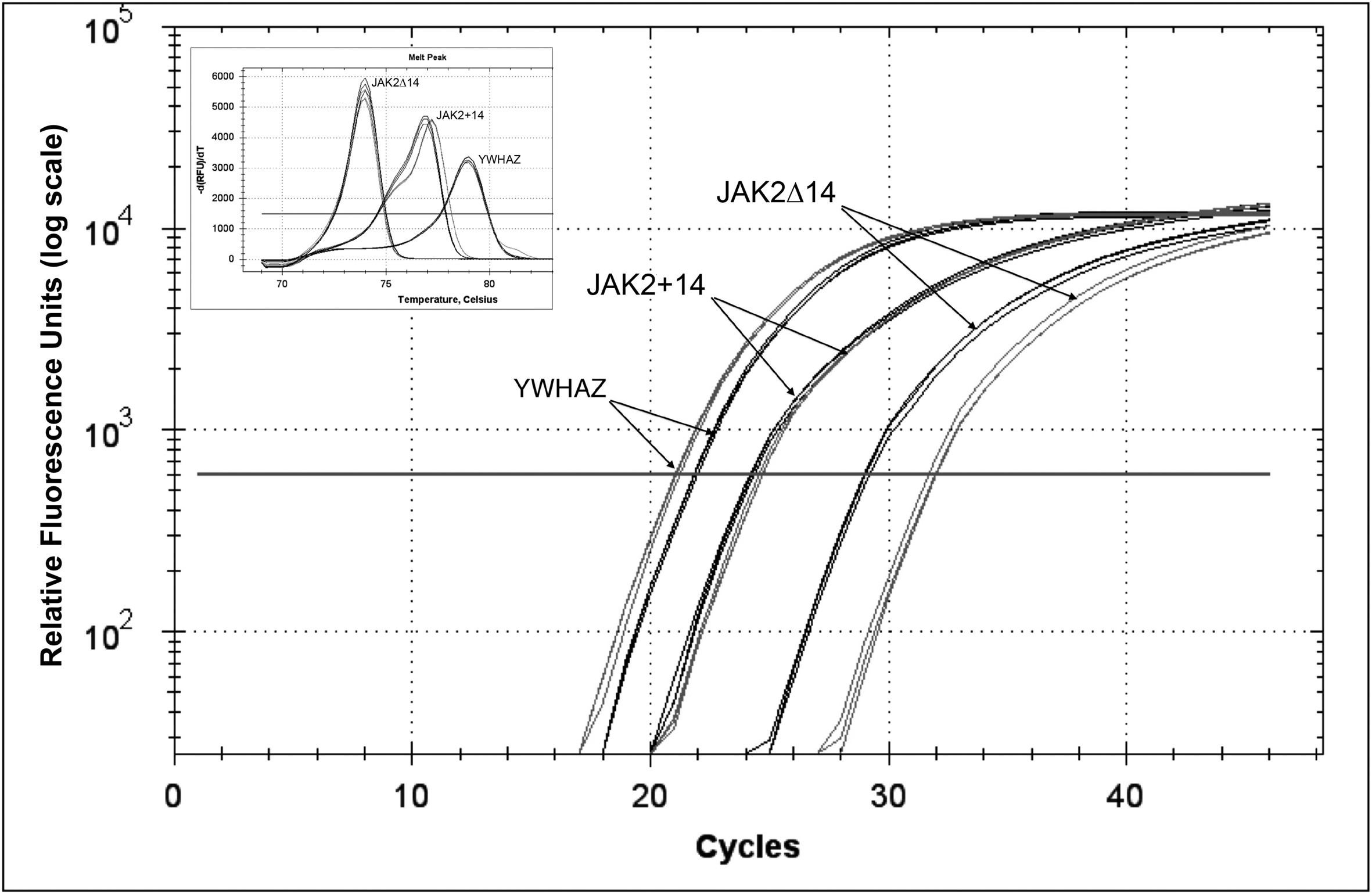

Supplement: S1 Fig — EvaGreen amplification signals for YWHAZ, JAK2+14 and JAK2Δ14, in two individuals with normal (grey) and increased (black) level of the exon 14-skipping isoform. Top left box shows melting peaks obtained by High Resolution Melting Analysis of the three amplification products: it can be observed the different melting peak morphology caused by the JAK2-V617F mutation present in the JAK2+14 transcripts of the patient with increased level of JAK2Δ14. (JPG) [file pone.0116636.s001.jpg]

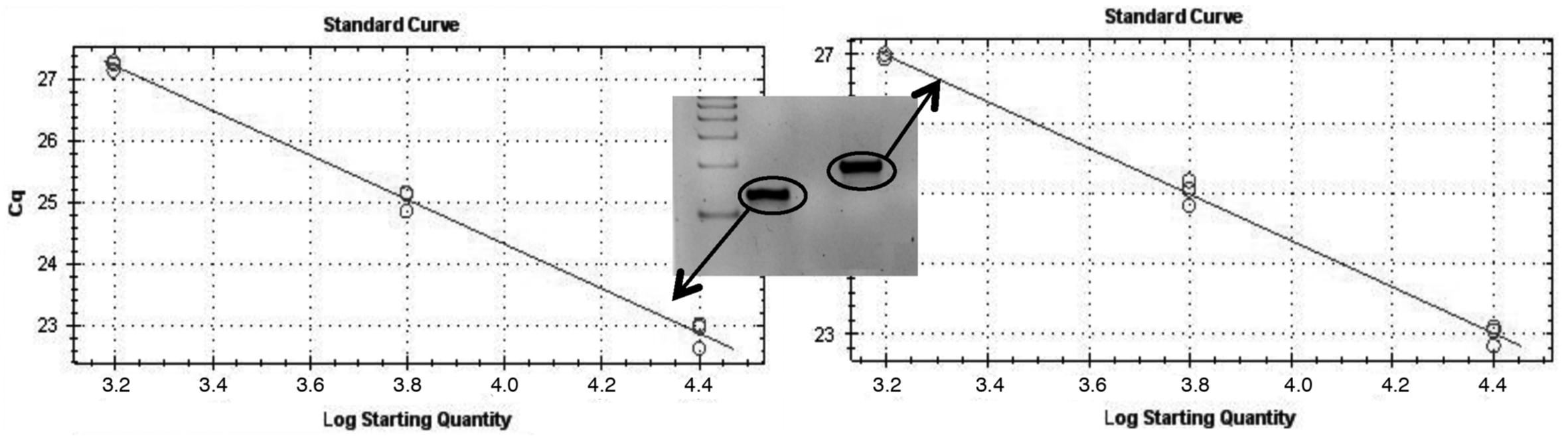

Supplement: S2 Fig — Equimolar dilutions of PCR-JAK2Δ14 (left) and PCR-JAK2+14 (right) amplicons, were used to generate two standard curves utilized to calculate the percentage of alternative transcript. The three points correspond to 1:4 serial dilutions of the gel-purified PCR products. (JPG) [file pone.0116636.s002.jpg]

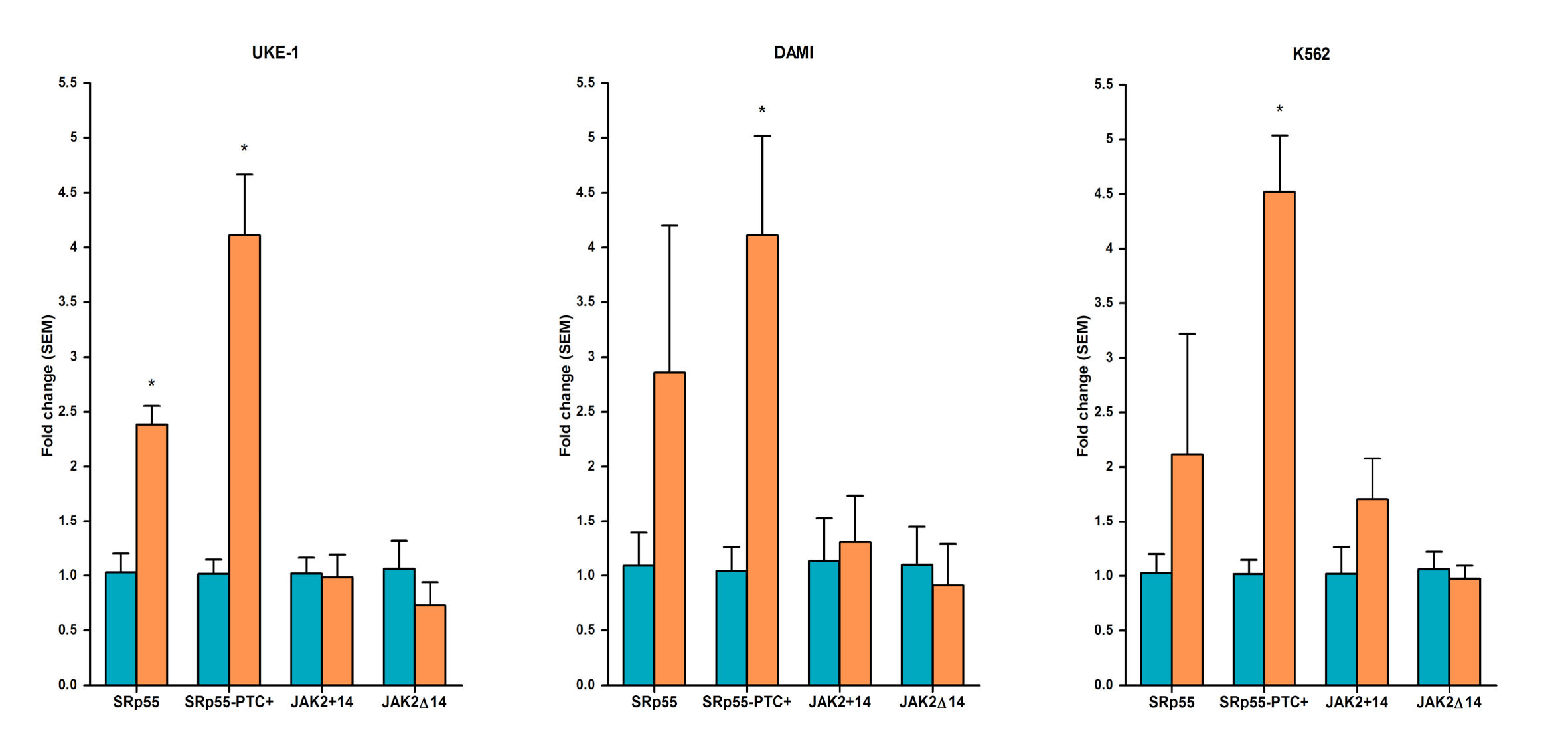

Supplement: S3 Fig — RT–qPCR was used to assay mRNAs levels in cell lines either homozygous for the JAK2-V617F mutation (UKE-1, DAMI) or wild type (K562). Transcript level ratios between CHX-treated (orange) and untreated cells (blue), are shown for: SRp55 constitutive transcript (SRp55), SRp55 PTC-containing isoform (SRp55-PTC+b), JAK2 full-length transcript (JAK2+14) and JAK2 exon 14 skipping isoform (JAK2Δ14). Data are expressed as means (± SEM) of three independent experiments performed using the same cell line. Normalized expression of targets genes was obtained using the two genes with the lowest geNorm M-value: YWHAZ/HPRT1 for DAMI, GAPDH/HPRT1 for K562 and UKE-1. Asterisks (*) indicate significant changes in gene expression after treatment. (JPG) [file pone.0116636.s003.jpg]

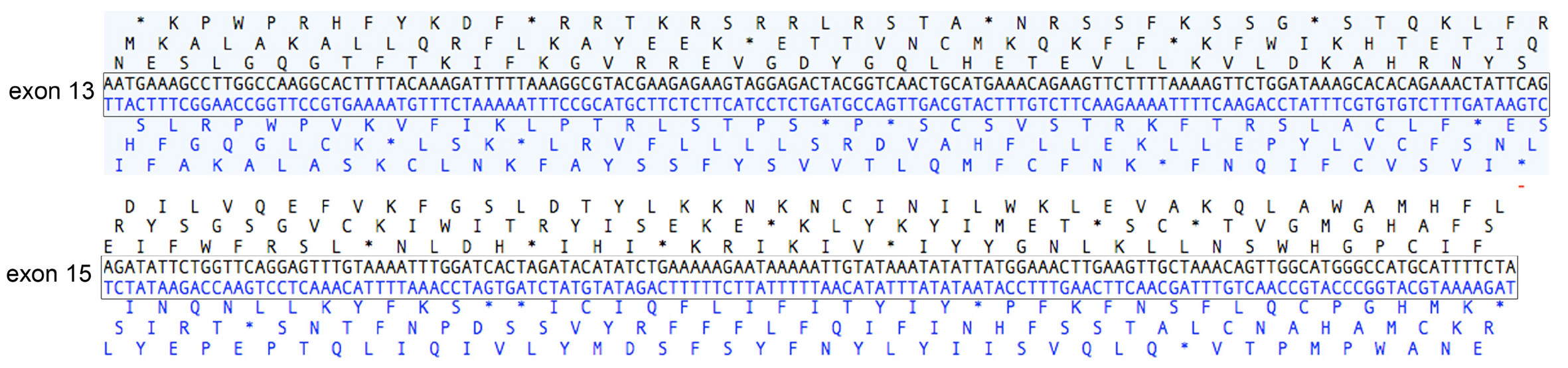

Supplement: S4 Fig — The sense strand (black), its complementary strand (blue) and their possible phases of translation, are shown. Single-letter code is used to represent the amino acids. A stop codon is indicated by an asterisk (*). The reading frame, used in the translation of the full-length transcript (JAK2+14), is represented in the first row above the sense strand. (JPG) [file pone.0116636.s004.jpg]

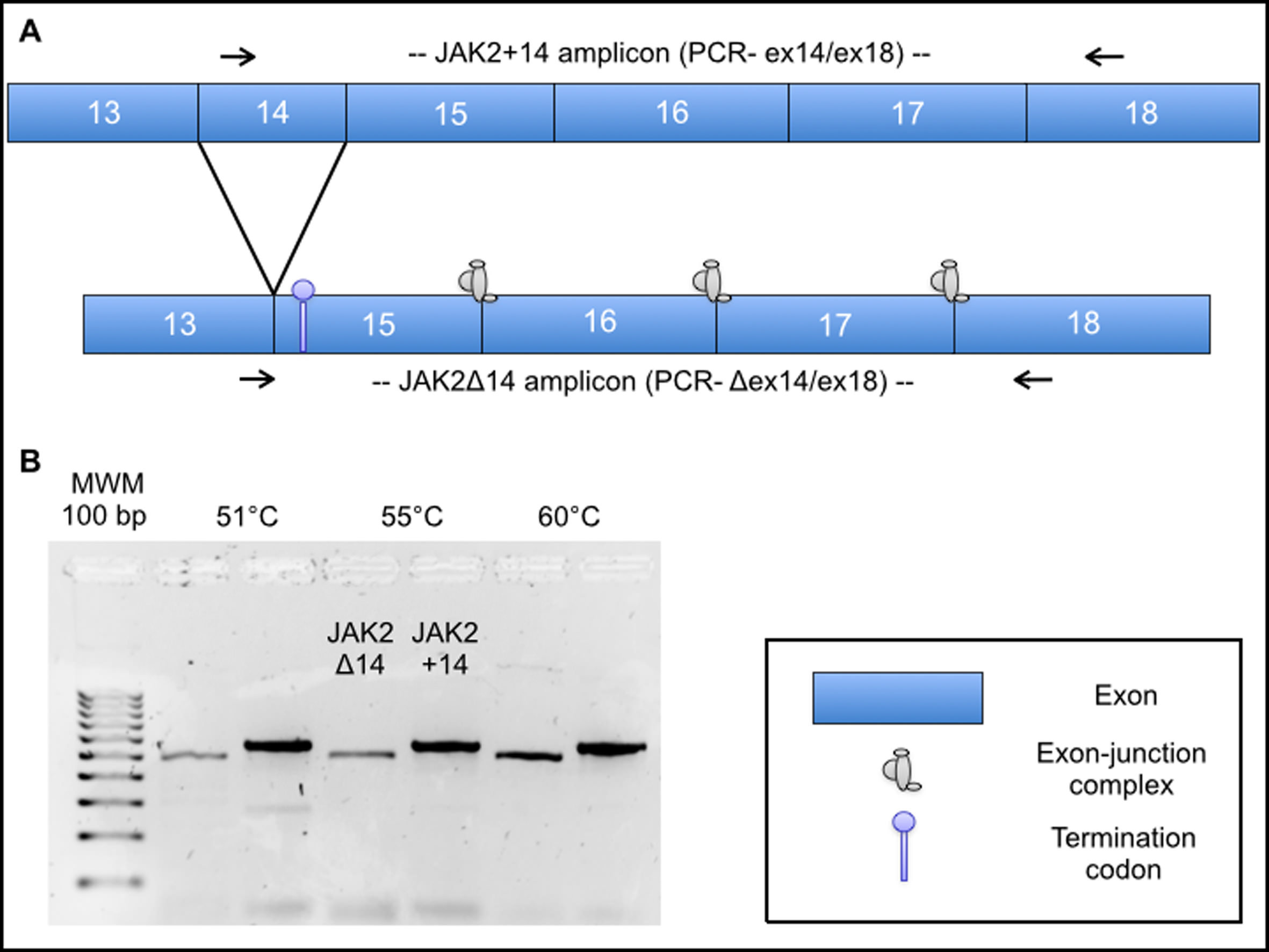

Supplement: S5 Fig — (A) The diagram shows the location of the primers in the JAK2 full-length mRNA (above) and in the isoform lacking exon 14 (below). As in the qPCR, forward primers were specific for each isoform while the reverse primer was, in both amplifications, localized in exon 18 (S2 Table). In the alternative isoform, the hypothetical position of the stop codon and exon junction complexes (which is expected to activate the NMD system), are indicated. (B) Electrophoresis of PCR products obtained by amplifying the cDNA of a patient with 2.5% level of JAK2Δ14 isoform, at three different annealing temperatures. The expected amplicon sizes are 495 bp for the JAK2Δ14 isoform (PCR-Δex14/ex18) and 556 bp for the JAK2+14 constitutive isoform (PCR-ex14/ex18). (JPG) [file pone.0116636.s005.jpg]
