# Supplementary material for: JAK2 Exon 14 Skipping in Patients with Primary Myelofibrosis: A Minor Splice Variant Modulated by the JAK2-V617F Allele Burden
Source: PLoS One. 2015 Jan 24;10(1):e0116636. doi: 10.1371/journal.pone.0116636 (PMC4305294; doi:10.1371/journal.pone.0116636)
Supplement: S1 Table — (PDF) [file pone.0116636.s006.pdf]

Table S1. RNA quality control assay results for seven randomly chosen cDNA samples used in this study.

	5'/3' ratio-mRNA integrity assay [A]			DNA contamination control assay [B]	Positive PCR control assay [C]
Sample	Cq (RQ1) [D]	Cq (RQ2) [D]	∆Cq (RQ2-RQ1)	Cq (gDNA) [D]	Cq (PCR) [D]
Control 1	15.03	14.52	- 0.51	No amplification	22.98
Control 2	15.03	15.20	0.17	No amplification	23.08
Patient 1	16.02	15.85	- 0.17	No amplification	22.91
Patient 2	15.74	15.91	0.17	35.17	23.16
Patient 3	14.75	14.26	-0.49	No amplification	22.89
Patient 4	15.01	14.52	-0.49	No amplification	22.82
UKE-1 cell line	18,29	17,49	-0.80	No amplification	22.92

[A] According to manufacturer instructions (Bio-Rad), if ∆Cq (RQ2-RQ1) is minor than 3, mRNA degradation is minimal and will likely have little to no effect on gene expression results.

[B] Cq>35 (threshold 300 Relative Fluorescence Units (RFU)) indicates single copy detection to no gDNA present.

[C] Cq>30 (threshold 300 RFU) indicates poor PCR performance due to the presence of inhibitors in RNA samples.

[D] Threshold = 300 RFU.
