# Supplementary material for: JAK2 Exon 14 Skipping in Patients with Primary Myelofibrosis: A Minor Splice Variant Modulated by the JAK2-V617F Allele Burden
Source: PLoS One. 2015 Jan 24;10(1):e0116636. doi: 10.1371/journal.pone.0116636 (PMC4305294; doi:10.1371/journal.pone.0116636)
Supplement: S2 Table — (PDF) [file pone.0116636.s007.pdf]

Table S2. Primers used in RT-qPCR experiments.

Amplicon name	GenBank accession number	Amplicon: length	Primer 5' position (exon)	Primer sequence
PCR-JAK2Δ14	N.A.	130 bp	2250 (13-15 junction)	GCACACAGAAACTATTCAGAGAT
			2467 (15)	GCCAACTGTTTAGCAACTTCA
PCR-JAK2+14	NM_004972.3	191 bp	2277 (14)	TTTGAAGCAGCAAGTATGATGAGC
			2467 (15)	GCCAACTGTTTAGCAACTTCA
YWHAZ[1]	NM_003406.3	94 bp	1145 (6)	ACTTTTGGTACATTGTGGCTTCAA
			1238 (6)	CCGCCAGGACAAACCAGTAT
GAPDH	NM_002046.3	130 bp	129 (2-3 junction)	CGGATTTGGTCGTATTGG
			258 (4)	GGTGGAATCATATTGGAACA
HPRT1 [1]	NM_000194.2	94 bp	578 (6)	TGACACTGGCAAAACAATGCA
			671 (6-7 junction)	GGTCCTTTTCACCAGCAAGCT
UBC	NM_021009.6	158 bp	447 (1-2 junction)	CGTCACTTGACAATGCAG
			604 (2)	TGTTTTCCAGCAAAGATCAG
SRp55 [2]	NM_006275.5	165 bp	55 (1)	CCCCTGTGGTGTGAGGCGCGTGTTC
			219 (1)	CCTTCTCCCGGACGTTGTAGCTCAG
Srp55-PTC+[2]	NR_034009.1	253 bp	412 (2-3 junction)	GCTACGGAAGCCGCATGACCAATGG
			664 (3)	GGCCACAAAACACGCAAGGTAACAG
hnRNP-A1	NM_002136.2	122 bp	544 (4)	GCTTTGCCTTTGTAACCTTTGACG
			665 (5)	AGCCATCTCTTGCTTTGACAGGG

1. Vandesompele J, De Preter K, Pattyn F, Poppe B, Van Roy N, et al. (2002) Accurate normalization of real-time quantitative RT-PCR data by geometric averaging of multiple internal control genes. Genome Biol 3: RESEARCH0034.

2. Lareau LF, Inada M, Green RE, Wengrod JC, Brenner SE (2007) Unproductive splicing of SR genes associated with highly conserved and ultraconserved DNA elements. Nature 446: 926–929. doi:10.1038/nature05676
