# Supplementary material for: JAK2 Exon 14 Skipping in Patients with Primary Myelofibrosis: A Minor Splice Variant Modulated by the JAK2-V617F Allele Burden
Source: PLoS One. 2015 Jan 24;10(1):e0116636. doi: 10.1371/journal.pone.0116636 (PMC4305294; doi:10.1371/journal.pone.0116636)
Supplement: S3 Table — (PDF) [file pone.0116636.s008.pdf]

Table S3. Primers used in RT-PCR experiments.

Amplicon name	GenBank accession number	Amplicon: length	Primer 5'position (exon)	Primer sequence
PCR-Δex14/ex18	N.A.	495 bp	2250 (13-15 junction)	GCACACAGAAACTATTCAGAGAT
			2832 (18)	ATTCTGCCCACTTTGGTG
PCR-ex14/ex18	NM_004972.3	556 bp	2277 (14)	TTTGAAGCAGCAAGTATGATGAGC
			2832 (18)	ATTCTGCCCACTTTGGTG
