# Supplementary material for: JAK2 Exon 14 Skipping in Patients with Primary Myelofibrosis: A Minor Splice Variant Modulated by the JAK2-V617F Allele Burden
Source: PLoS One. 2015 Jan 24;10(1):e0116636. doi: 10.1371/journal.pone.0116636 (PMC4305294; doi:10.1371/journal.pone.0116636)
Supplement: S4 Table — Twelve different matrices implemented in Human Splicing Finder (HSF) 3.0, are presented. (PDF) [file pone.0116636.s009.pdf]

Table S4. Identification of potential splicing regulatory sequences in wild type and V617F mutated *JAK2* exon 14 sequences. Twelve different matrices implemented in Human Splicing Finder (HSF) 3.0, are presented.

Matrice	Type of exonic element	Position in exon 14 sequence (88 nucleotides)	Sequence motif (WT)	Sequence motif (mutant)	Variation
1) ESE finder	ESE	70	TGTGTC<br>(Linked protein: SRp55)	TGTTTCTG<br>(Linked protein: SC35)	**Site broken/New site**
2) EIEs [a] [3]	ESE	68	TATGTG	-	**Site broken**
3) ESR$_s$ [b] [4]	ESE/ESS	70	TGTGTC	-	**Site broken**
4) RESCUE-ESE[c] hexamers [5]	ESE	-	-	-	No difference
5) Enhancers motifs[d] [6]	ESE	-	-	-	No difference
6) PESE [e] octamers [7]	ESE	-	-	-	No difference
7) FAS-ESS hexamers[f] [8]	ESS	70<br>71<br>73	-	TGTTTC/<br>GTTTCT/<br>TTCTGT	**New site**
8) FAS-ESS decamers[f] [8]	ESS	-	-	-	No difference
9) PESS[g] octamers [7]	ESS	71	-	GTTTCTGT	**New site**
10) IIEs[h] [3]	ESS	68<br>69<br>70<br>71<br>72<br>73	TATGTG<br>ATGTGT<br>TGTGTC<br>GTGTCT<br>TGTCTG<br>GTCTGT	TATGTT<br>ATGTTT<br>TGTTTC<br>GTTTCT<br>TTTCTG<br>TTCTGT	No difference
11) hnRNP motifs[i] [6]	ESS<br>(Linked factor: hnRNP-A1)	68	TATGTG<br>(score: 66.19%)	TATGTT<br>(score: 68.10%)	**Variation: 2.88%**
12) Silencer motifs (Sironi et al.) [9]	ESS	-	-	-	No difference

[a] Exon Identity Elements;
[b] Exonic Splicing Regulatory Sequences (ESE or ESS depending on exonic position);
[c] Hybrid computational/experimental method;
[d] Human Splice Finder matrice based on experimental data;
[e] Predicted Exonic Splicing Enhancers;
[f] Fluorescence-Activated Screen for Exonic Splicing Silencers;
[g] Predicted Exonic Splicing Silencers;
[h] Intron Identity Elements;
[i] Human Splice Finder matrice based on experimental data (hnRNP-A1 threshold: 65.476%).

REFERENCES

1. Vandesompele J, Preter KD, Pattyn F, Poppe B, Roy NV, et al. (2002) Accurate normalization of real-time quantitative RT-PCR data by geometric averaging of multiple internal control genes. Genome Biol 3: research0034.1.

2. Lareau LF, Inada M, Green RE, Wengrod JC, Brenner SE (2007) Unproductive splicing of SR genes associated with highly conserved and ultraconserved DNA elements. Nature 446: 926–929. doi:10.1038/nature05676

3. Zhang C, Li W-H, Krainer AR, Zhang MQ (2008) RNA landscape of evolution for optimal exon and intron discrimination. Proc Natl Acad Sci USA 105: 5797–5802. doi:10.1073/pnas.0801692105

4. Goren A, Ram O, Amit M, Keren H, Lev-Maor G, et al. (2006) Comparative analysis identifies exonic splicing regulatory sequences--The complex definition of enhancers and silencers. Molecular Cell 22: 769–781. doi:10.1016/j.molcel.2006.05.008

5. Fairbrother WG, Yeh R-F, Sharp PA, Burge CB (2002) Predictive identification of exonic splicing enhancers in human genes. Science 297: 1007–1013. doi:10.1126/science.1073774

6. Desmet FO, Hamroun D, Lalande M, Collod-Beroud G, Claustres M, et al. (2009) Human Splicing Finder: an online bioinformatics tool to predict splicing signals. Nucleic Acids Res 37: e67–e67. doi:10.1093/nar/gkp215

7. Zhang XH-F, Chasin LA (2004) Computational definition of sequence motifs governing constitutive exon splicing. Genes Dev. 18: 1241–1250. doi:10.1101/gad.1195304

8. Wang Z, Rolish ME, Yeo G, Tung V, Mawson M, et al. (2004) Systematic identification and analysis of exonic splicing silencers. Cell 119: 831–845. doi:10.1016/j.cell.2004.11.010

9. Sironi M, Menozzi G, Riva L, Cagliani R, Comi GP, et al. (2004) Silencer elements as possible inhibitors of pseudoexon splicing. Nucleic Acids Res 32: 1783–1791. doi:10.1093/nar/gkh341
